# Right Atrial Thrombus in a COVID-19 Child Treated Through Cardiac Surgery

**DOI:** 10.3389/fcvm.2020.579522

**Published:** 2020-11-12

**Authors:** Hamid Bigdelian, Mohsen Sedighi, Mohammad Reza Sabri, Bahar Dehghan, Zahra Pourmoghaddas, Rana Saleh, Alireza Sherafat, Gholamreza Massoumi, Mohammad Kazem Rezaei, Masoud Shahbazi

**Affiliations:** ^1^Cardiovascular Research Institute, Isfahan University of Medical Sciences, Isfahan, Iran; ^2^Neuroscience Research Center, Iran University of Medical Sciences, Tehran, Iran; ^3^Pediatric Department, School of Medicine, Isfahan University of Medical Sciences, Isfahan, Iran; ^4^School of Medicine, University of Central Lancashire, Preston, United Kingdom

**Keywords:** COVID-19, cardiac surgery, pediatric, thrombus—echocardiography, fever

## Abstract

We herein report a case of large intracardiac thrombus in a child with SARS-CoV-2 infection (COVID-19). The diagnosis of COVID-19 was confirmed through HRCT and RT-PCR. Transthoracic echocardiography revealed a large thrombus in the right atrium treated successfully *via* cardiac surgery. The underlying mechanisms of this thrombus in the COVID-19 infection may be attributed to the hypercoagulation and inflammatory condition incurred by the COVID-19 virus.

## Introduction

In December 2019, an outbreak of new viral pneumonia caused by the novel coronavirus (SARS-CoV-2) occurred in Wuhan, China (1). This virus is responsible for COVID-19 (Corona Virus Disease 2019) and can lead to different symptoms ranging from a mild viral disease to acute respiratory distress syndrome (ARDS), multi-organ failure, and death (2, 3). Although cardiovascular complications of COVID-19 has been well-described in the current literature, intracardiac thrombus complication in children has been rarely reported. Intracardiac thrombus caused by SARS-CoV-2 infection is a serious and life-threatening complication in COVID-19 patients (4) and the size of thrombus at the time of diagnosis correlates strongly with increased risk of thromboembolism and sudden death (5). Here, we describe a case report of large thrombus formation in the right atrium (RA) of a child with COVID-19 infection required emergency cardiac surgery, as the only preferred treatment to remove such a large thrombus.

## Case Report

**Figure 1:**
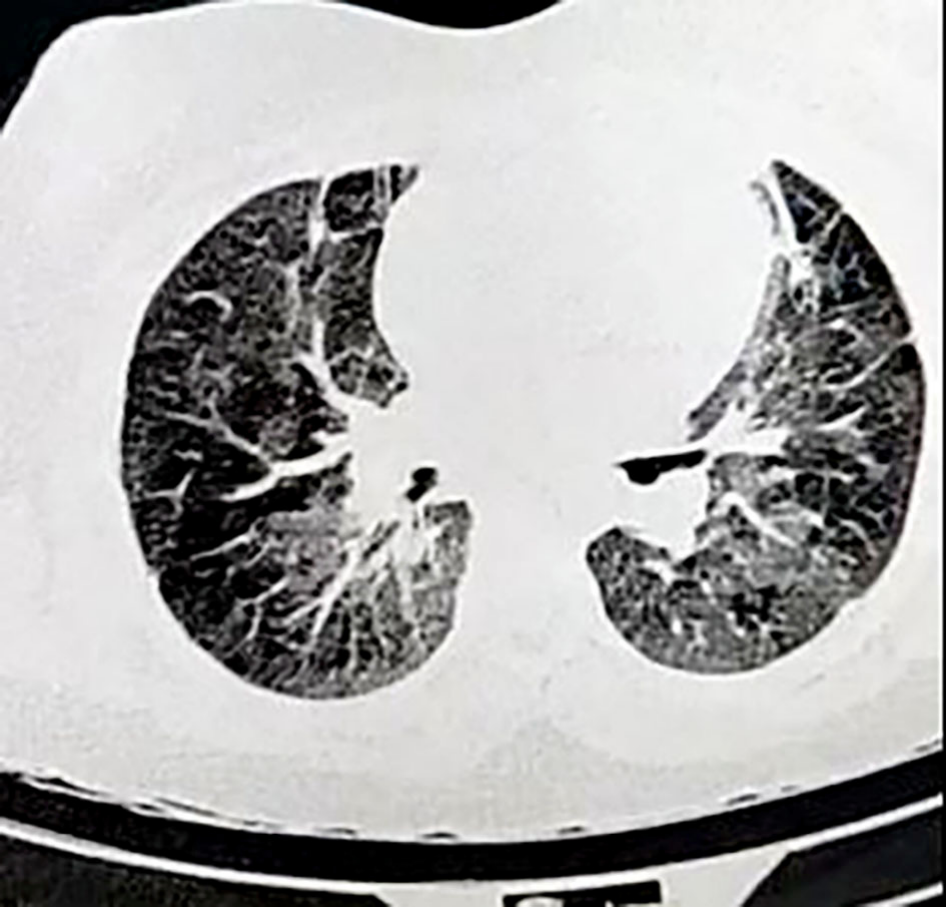
High-resolution computed tomography (HRCT) of the lungs in COVID-19 child showing bilateral multiple patchy areas of ground glass opacities.

**Figure 2:**
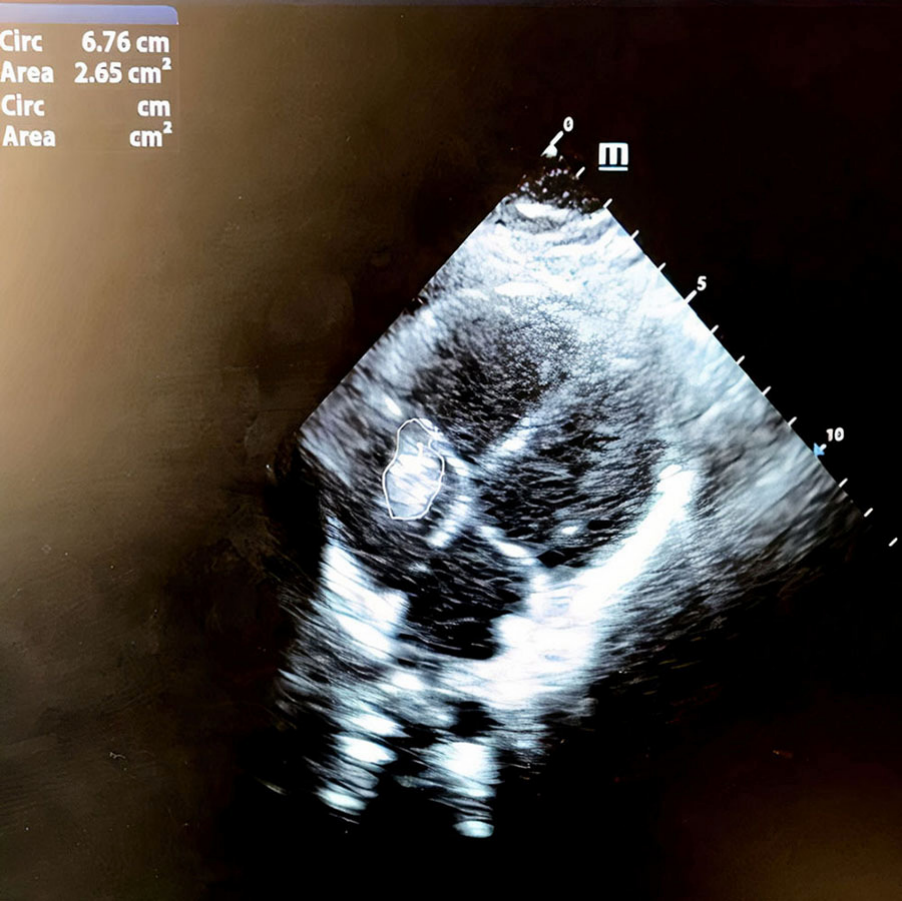
Transthoracic echocardiogram showing a large 2.5 × 1.5 cm mass on tricuspid valve leaflet protruded to the right atrium and right ventricle.

**Figure 3:**
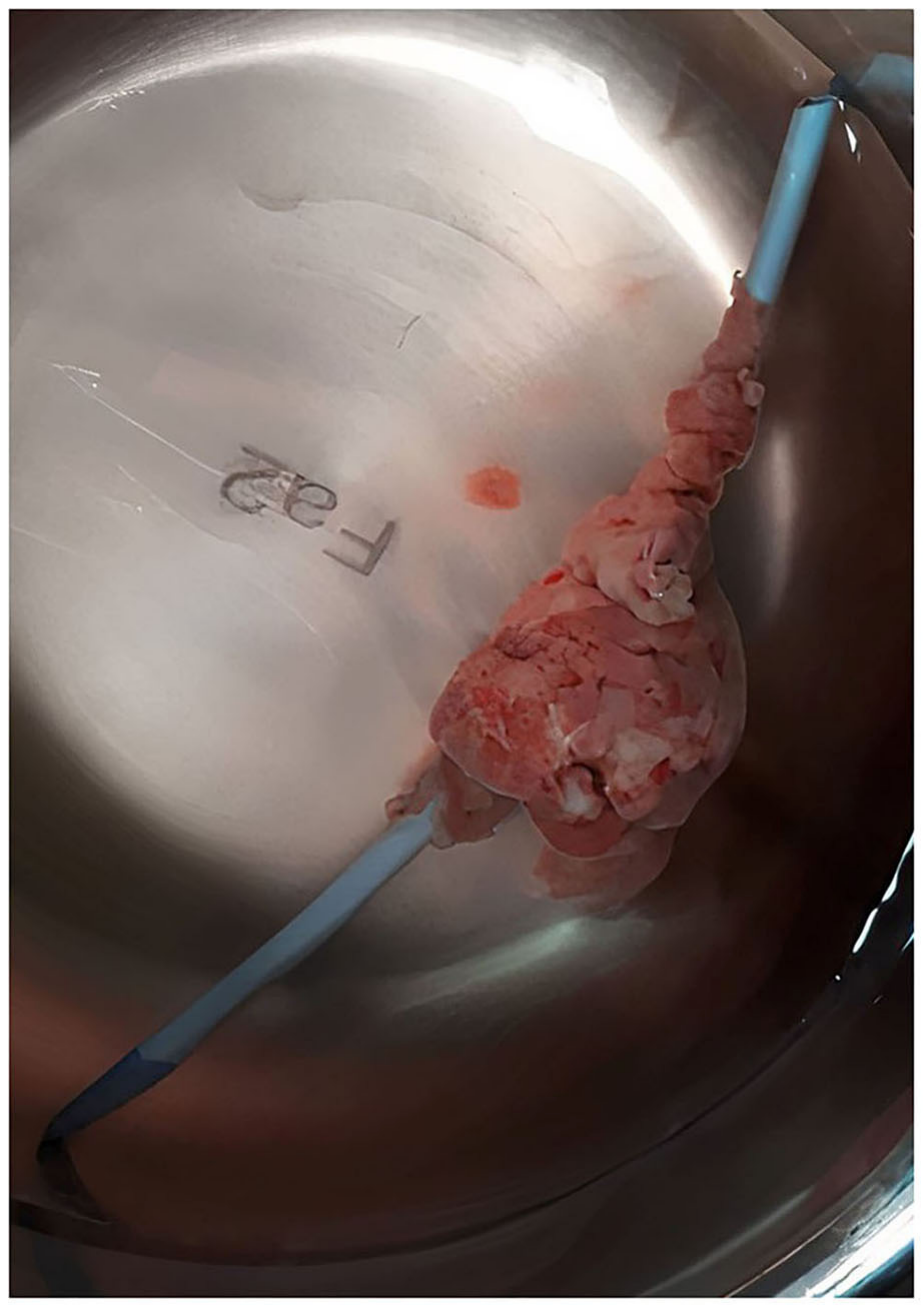
Formation of a large thrombus around the CVC that was removed along with catheter via open heart surgery.

On 7 April 2020, an 11-years-old boy was admitted to the pediatric hospital in Esfahan with high fever, dyspnea, and skin rashes. Past medical history of patient showed that he was a known case of seizure disorder and neurodevelopmental delay, and had been under treatment with phenobarbital. Blood gas analysis on the admission time showed a pH of 7.27, pCO_2_ of 49.5 mmHg, pO_2_ of 44.8 mmHg, and oxygen saturation (O_2_ Sat) of 73%. Also, his laboratory test results were as follows: white blood cell count (WBC) of 11.4 × 10^*^9/L (reference range: 4.5–11), C-reactive protein (CRP) 38 mg/dL (reference range: up to 6), erythrocyte sedimentation rate (ESR) 38 mm/h (reference range: up to 20), and lactate dehydrogenase (LDH) 427 U/L (reference range: 140–280). Chest x-ray and high-resolution computed tomography (HRCT) showed typical ground-glass opacitiesin both lungs, suggesting viral pneumonia (Figure 1). Throat swab samples analysis by reverse transcription-polymerase chain reaction (RT-PCR) confirmed COVID-19 infection and treatment of disease was started according to the Iranian pediatric protocol including hydroxychloroquine (5 mg/kg/dose), lopinavir /ritonavir (230 mg/m^2^/dose), ceftriaxone (75 mg/kg/dose), and vancomycin (10 mg/kg/dose) (6). On the 2nd day of hospitalization, he presented shortness of breathing and decreased level of consciousness (LOC) that led to intubation and he put on a ventilator. After extubation, Bi-level positive airway pressure (BiPAP) was administrated for respiratory support. Despite receiving drug treatment for COVID-19, his laboratory test results were significant for continuous leukocytosis and neutrophilia (Table 1). Because of persistent fever and tachycardia, pediatric cardiology consultation was requested and transthoracic echocardiography revealed a large mobile homogenous mass (2.5 × 1.5 cm) on the tricuspid valve leaflet extended to the RA and right ventricle (RV) with attachment to the tip of central venous catheter (CVC) that was in favor of thrombus or vegetation (Figure 2). Besides, trivial tricuspid regurgitation (TR) and trivial pulmonary insufficiency (PI) were found. Consequently, he was transferred to the Chamran Heart Center on 16 April 2020 for surgical intervention. A sternotomy with cardiopulmonary bypass was performed and intraoperative observations revealed a large thrombus in RA which was removed completely together with CVC while cardiac valves were preserved (Figure 3). After the surgery, he was admitted to the ICU, and the pediatric ward thereafter. Culture result of the thrombus showed no fungal and bacterial infection and also SARS-CoV-2 RNA was not detected in the mass. Histological analysis of the mass showed inflammatory infiltrate (mostly neutrophils) formation along with organizing thrombus and partial necrosis of tricuspid valve leaflet. He was discharged after 2 weeks in good health condition.

**Table 1:** Laboratory test results of patient during the course of disease.

**Variables**	**Reference range**	**Admission**	**Day 5**	**Day 10**	**Before surgery**	**After surgery**
RBC count (× 10^*^12/L)	4.5–6.5	3.84	4.04	4.13	3.88	4.63
Hematocrit (%)	41–51	36.9	38.3	38.4	31.6	41.3
Hemoglobin (g/dL)	13–17	11.5	12.5	13.3	10.9	14.5
WBC (× 10^*^9/L)	4.5–11	11.4	12.2	13.5	21.3	11.2
Lymphocyte count (%)	20–40	27	15	16	15.1	17.5
Neutrophil count (%)	50–70	66.4	78	72.1	75.7	72.9
Platelet count (× 10^*^9/L)	150–450	252	279	247	400	238
PT (second)	11–13	13	12.8	12.2	17.1	16
PTT (second)	26–45	38	37	33	30	28
INR	0.8–1.2	1.1	1.1	1.1	1.4	1.2
Blood culture (×3)	+ / -	-	-	-	-	-

*RBC, red blood cell; WBC, white blood cell; PT, prothrombin time; PTT, partial thromboplastin time; INR, international normalized ratio*.

## Discussion

Accumulating evidence has indicated cardiovascular involvement of COVID-19 infection which can lead to the poor clinical prognosis of this disease. Discriminating between a cardiac or pulmonary cause of symptoms can be difficult because each may present mostly with dyspnea. Recent reports have shown that COVID-19 is associated with the increased incidence of cardiovascular complications including myocarditis, acute cardiac injury, and arrhythmias (7). Moreover, it has been demonstrated that inflammation and thrombosis mutually reinforce each other and COVID-19 infection can lead to the coagulopathy likely due to infection-induced inflammatory changes (8). Remarkable inflammation is observed in patients with COVID-19 infection presenting by elevated levels of IL-6, increased CRP and erythrocyte sedimentation rate (ESR). This inflammatory condition and subsequent activation of coagulation are the probable causes for the hypercoagulable state in COVID-19 infection (9). The intracardiac thrombus formation has rarely been described in the COVID-19 patients and our finding supports current reports that indicate higher thrombotic risk in COVID-19 patients (10, 11). The presence of a large thrombus in RA was an unexpected finding in our case because he had no history of heart disease or coagulopathy. Similar to the other reports, we believe that the bigger the size of the clot, the greater the chance that medical treatment will fail (12). Removal of such a large thrombus needs surgical intervention and surgical thrombectomy should be opted to treat large right atrial thrombus (more than 2 cm) in the absence of any contraindication for surgery. In parallel with us, a literature review by Negulescu et al. has reported a lower mortality rate in the surgical intervention compared to the anticoagulation treatment. Meanwhile, anticoagulation treatment correlates with the theoretical risk of lysed clots lodging in pulmonary arteries and consequent pulmonary thromboembolism (13). In conclusion, there is an increasing concern about hypercoagulation and acute thrombosis in patients with COVID-19 infection. Therefore, conservative treatment with anticoagulation along with vigilant observation is recommended in all COVID-19 patients to prevent subsequent hypercoagulation and thrombus formation.

## Data Availability Statement

The raw data supporting the conclusions of this article will be made available by the authors, without undue reservation.

## Ethics Statement

This study involving human participant was reviewed and approved by Isfahan University of Medical Sciences (IR.MUI.MED.REC.1399.198). Written informed consent to participate in this study was provided by the participants' legal guardian/next of kin. Written informed consent was obtained from the individual(s), and minor(s)' legal guardian/next of kin, for the publication of any potentially identifiable images or data included in this article.

## Author Contributions

All authors contributed to the analysis, interpretation of data, wrote the manuscript, approved the final version of the manuscript, and agreed to be accountable for all aspects of the work.

## Conflict of Interest

The authors declare that the research was conducted in the absence of any commercial or financial relationships that could be construed as a potential conflict of interest.
